# Seafood Paramyosins as Sources of Anti-Angiotensin-Converting-Enzyme and Anti-Dipeptidyl-Peptidase Peptides after Gastrointestinal Digestion: A Cheminformatic Investigation

**DOI:** 10.3390/molecules27123864

**Published:** 2022-06-16

**Authors:** Tsun-Thai Chai, Clara Chia-Ci Wong, Mohamad Zulkeflee Sabri, Fai-Chu Wong

**Affiliations:** 1 Department of Chemical Science, Faculty of Science, Universiti Tunku Abdul Rahman, Kampar 31900, Malaysia; clara2000genesis@1utar.my (C.C.-C.W.); wongfc@utar.edu.my (F.-C.W.); 2 Center for Agriculture and Food Research, Universiti Tunku Abdul Rahman, Kampar 31900, Malaysia; 3 Green Chemistry and Sustainable Technology Cluster, Bioengineering Section, Malaysian Institute of Chemical and Bioengineering Technology, Universiti Kuala Lumpur, Lot 1988, Bandar Vendor Taboh Naning, Alor Gajah 78000, Malaysia; mzulkeflee@unikl.edu.my

**Keywords:** anti-ACE, anti-DPP-IV, gastrointestinal digestion, in silico, molecular docking, molecular dynamics, paramyosin, pharmacokinetics, seafood, target fishing

## Abstract

Paramyosins, muscle proteins occurring exclusively in invertebrates, are abundant in seafoods. The potential of seafood paramyosins (SP) as sources of anti-angiotensin-converting-enzyme (ACE) and anti-dipeptidyl-peptidase (DPP-IV) peptides is underexplored. This in silico study investigated the release of anti-ACE and anti-DPP-IV peptides from SP after gastrointestinal (GI) digestion. We focused on SP of the common octopus, Humboldt squid, Japanese abalone, Japanese scallop, Mediterranean mussel, Pacific oyster, sea cucumber, and Whiteleg shrimp. SP protein sequences were digested on BIOPEP-UWM, followed by identification of known anti-ACE and anti-DPP-IV peptides liberated. Upon screening for high-GI-absorption, non-allergenicity, and non-toxicity, shortlisted peptides were analyzed via molecular docking and dynamic to elucidate mechanisms of interactions with ACE and DPP-IV. Potential novel anti-ACE and anti-DPP-IV peptides were predicted by SwissTargetPrediction. Physicochemical and pharmacokinetics of peptides were predicted with SwissADME. GI digestion liberated 2853 fragments from SP. This comprised 26 known anti-ACE and 53 anti-DPP-IV peptides exhibiting high-GI-absorption, non-allergenicity, and non-toxicity. SwissTargetPrediction predicted three putative anti-ACE (GIL, DL, AK) and one putative anti-DPP-IV (IAL) peptides. Molecular docking found most of the anti-ACE peptides may be non-competitive inhibitors, whereas all anti-DPP-IV peptides likely competitive inhibitors. Twenty-five nanoseconds molecular dynamics simulation suggests the stability of these screened peptides, including the three predicted anti-ACE and one predicted anti-DPP-IV peptides. Seven dipeptides resembling approved oral-bioavailable peptide drugs in physicochemical and pharmacokinetic properties were revealed: AY, CF, EF, TF, TY, VF, and VY. In conclusion, our study presented in silico evidence for SP being a promising source of bioavailable and safe anti-ACE and anti-DPP-IV peptides following GI digestions.

## 1. Introduction

Bioactive peptides, especially those derived from dietary sources, are short fragments of food proteins that exert physiologically relevant activities. Such peptides, frequently 2–20 residues in length, could be liberated from food proteins by means of chemical or enzymatic hydrolysis, microbial fermentation, but also naturally in vivo during gastrointestinal (GI) digestion. The past ten years have seen a drastic surge in research exploring food-derived bioactive peptides. Such investigations have led to the discovery of numerous peptides that exert diverse bioactivities, encompassing antihypertension, antidiabetic, antioxidant, and anticancer activities. A key driver behind such intensive explorations is the recognition that that such peptides could have potential applications as nutraceuticals/functional food ingredients and therapeutic/prophylactic agents [1,2,3,4].

Traditionally, bioactive peptide discovery is mainly driven by wet-lab research, often involving the time-consuming process of isolating proteins from chosen samples, release of peptides from food proteins, bioactivity-guided purification of protein hydrolysates, mass spectrometric identification of peptides, synthesis of peptides, and lastly, validation of peptide bioactivity [1,5]. However, the in silico approach is increasingly embraced by researchers in bioactive peptide discovery due to its low cost and efficiency in peptide screening. Some studies have focused on only an in silico approach; others have integrated in silico analysis into their wet-lab experimentations. The toolbox for in silico bioactive peptide discovery encompasses, among others, various online servers, cheminformatics tools, simulation and visualization software, and bioactive peptide databases [6,7]. In this computational study, we adopted the in silico approach to screen for anti-angiotensin-converting-enzyme (ACE) and anti-dipeptidyl peptidase IV (DPP-IV) peptides released from seafood paramyosins following in silico GI digestion. ACE is a key player in the renin-angiotensin system, a pathway for the regulation of blood pressure in vivo. ACE inhibitors (e.g., Captopril) can help maintain normal blood pressure and thus can be used as antihypertensive drugs [8,9]. On the other hand, DPP-IV inhibitors improve the control of blood sugar levels in type 2 diabetes mellitus [10]. Inhibitors of DPP-IV (e.g., Anagliptin) can be used as oral antidiabetic drugs [11]. In this study, we also attempted to screen for bifunctional peptides exhibiting both anti-ACE and anti-DPP-IV activities. Such bifunctional peptides are valuable particularly in addressing complex pathological conditions (e.g., co-occurrence of high blood pressure in patients experiencing type 2 diabetes mellitus) [12].

Paramyosins are muscle proteins that occur exclusively in invertebrates, absent in vertebrate muscles. Paramyosins are enriched with about 20% glutamic acid residues. Paramyosin contents in scallop, squid, and oysters are 3, 14, and 19%, respectively. Notably, in the white adductor muscle of oysters and clams, paramyosins may comprise 38–48% of the total myofibrillar protein [13]. Despite their uniqueness and abundance in seafood invertebrates, there is very little information about seafood paramyosins as sources of bioactive peptides. A recent in silico investigation on the Portuguese oyster (*Crassostrea angulata*) found that paramyosin isoform X2 of the species could be a source of hundreds of anti-ACE (294) and anti-DPP-IV (517) peptides [14]. Thus, we hypothesized that other seafood paramyosins may also be sources of anti-ACE and anti-DPP-IV. In this in silico study, we focused on the paramyosins of eight species: the common octopus, Humboldt squid, Japanese abalone, Japanese scallop, Mediterranean mussel, Pacific oyster, sea cucumber, and Whiteleg shrimp, which are widely consumed worldwide. By virtually screening for anti-ACE and anti-DPP-IV peptides liberated from the paramyosins, we aimed to not only fill gaps of knowledge in the literature. Importantly, promising paramyosins that can be prioritized in future research as sources of nutraceuticals/drug candidates targeting hypertension and diabetes would be pinpointed. Mechanistic information on peptide-enzyme interactions as well as pharmacokinetics and drug-likeness of the peptides would also be explored.

## 2. Results and Discussion

### 2.1. Seafood Paramyosins

Nine paramyosin protein sequences were retrieved from UniProtKB (Table 1). One paramyosin sequence was found for each seafood species, except for the common octopus (CO), for which two isoforms (CO-X1 and CO-X2) were found. The paramyosins of the seafoods ranged from 516 residues (CO) to 934 residues (Japanese scallop, JS). Similarly, paramyosin isoform X2 of the common octopus (CO-X2) has the smallest molecular mass (59 kDa), whereas paramyosin of JS has the largest (107.5 kDa).

### 2.2. In Silico GI Digestion

**Figure 1:**
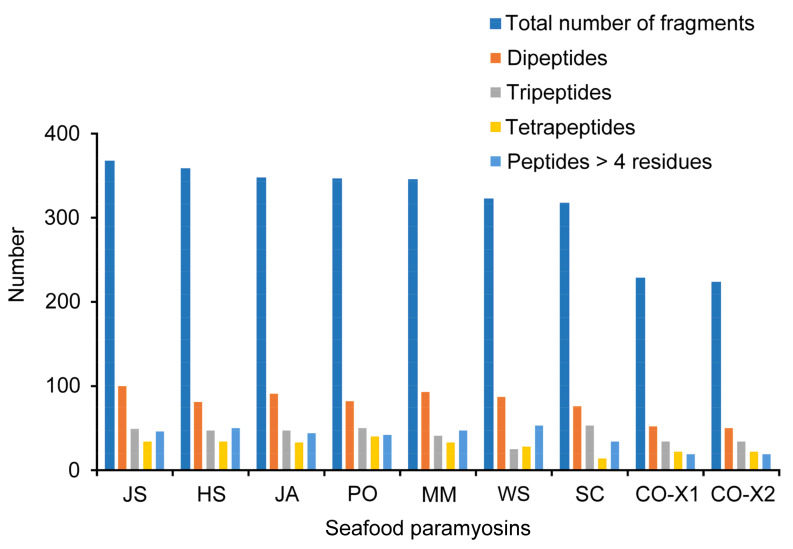
Distribution of peptides of different lengths released by in silico GI digestion of seafood paramyosins. An individual amino acid released from in silico GI digestion was counted as one fragment.

The in silico GI digestion of the nine paramyosins in Table 1 resulted in the release of 2853 peptide fragments. The outcome of the in silico hydrolysis is presented in Figure 1. Among the 2853 fragments liberated by the nine paramyosins, 1706 of them comprised two or more residues. In this study, we paid special attention to short peptides of several residues rather than the free amino acids since digested proteins are absorbed predominantly in the form of di- and tri-peptides, rather than individual amino acids [15,16,17,18]. More than 300 peptide fragments were liberated from each protein, except for the two paramyosin isoforms of the common octopus (CO-X1; CO-X2). The paramyosin of JS, which has the largest number of residues (Table 1), liberated the largest number of fragments (367). CO-X2, the shortest among the nine paramyosins, released the lowest number of fragments (223). With the exceptions of CO-X1 and CO-X2, the other seafood paramyosins each potentially liberated more than 100 peptide fragments collectively as di- and tripeptides. Numerous such short peptides are known for ready uptake by the human intestinal cells, a process mediated by PepT1, a proton-coupled oligopeptide cotransporter [19]. Thus, whether such peptides exhibit any health-promoting effects, particularly anti-ACE and anti-DPP-IV activities, is of great interest. Dipeptides consistently formed the major group of short peptide fragments released from the nine paramyosins, ranging from 27.3% in JS to 22.4% in CO-X2. The paramyosin of JS also released the largest number of dipeptides (100) following in silico GI digestion, whereas CO-X2 released the fewest (50) (Figure 1). In contrast to the dipeptide group, peptide fragments > 4 residues long formed only 8.3% to 16.5% of the total pool of fragments released from the seafood paramyosins. The longest peptide fragment released was an 18-residue peptide originating from JS (Data not shown). Overall, our observations agree with that previously reported [20] where more peptide fragments were liberated from housefly larval proteins of larger peptide lengths. Our results also suggests that among the nine seafood paramyosins, the one from JS likely has the greatest number of pepsin-, trypsin-, and chymotrypsin cleavage sites in its sequence.

### 2.3. Screening for Anti-ACE and Anti-DPP-IV Peptides

A search against the BIOPEP-UWM database where experimentally validated bioactive peptides were deposited [21] revealed that 92 and 174 seafood paramyosin-derived peptides are known anti-ACE and anti-DPP-IV peptides, respectively. This implies that when ingested and digested in the GI tract, the seafood paramyosins are better as sources of anti-DPP-IV peptides than as sources of anti-ACE peptides. Nevertheless, not all such peptides are promising bioavailable anti-ACE and anti-DPP-IV agents. For example, ASL, ITF, and IVR are three known anti-ACE peptides released from the paramyosins following in silico GI digestion. The three peptides were predicted as having only low GI absorption by SwissADME (data not shown). Furthermore, molluscan paramyosins such as those from the common octopus and the Mediterranean mussel have been connected to food allergies [22]. Therefore, whether the peptide fragments released from the paramyosins following GI digestion is safe and easily absorbed by the GI tract is a pertinent issue. Thus, to better explore the potential of the nine seafood paramyosins as sources of orally available anti-ACE and anti-DPP-IV agents, we virtually screened the 266 known anti-ACE and anti-DPP-IV peptides for high GI absorption, non-allergenicity and non-toxicity. 

### 2.4. Screening for High GI Absorption, Non-Allergenicity, and Non-Toxicity

Twenty-six of the ninety-two known anti-ACE peptides (28.3%) liberated from the nine paramyosins through in silico GI digestion are potentially highly-absorbed by the human GI tract, non-allergenic, and non-toxic (Table 2). By contrast, 53 of the 174 known anti-DPP-IV peptides (30.5%) released from the paramyosins were likely to exhibit high GI absorption, non-allergenicity, and non-toxicity. By comparison, the nine paramyosins are a more promising source of potentially bioavailable and safe anti-DPP-IV peptides versus anti-ACE peptides. As shown in Table 2, WS was the most promising source of high-GI-absorption, non-allergenic, and non-toxic anti-DPP-IV peptides. At the individual paramyosin’s level, repeated sequences are found. For example, the 12 WS-derived anti-DPP-IV peptides can be consolidated into six unique sequences (IL, SL, TF, TY, VL, and VY). Across the different seafood species, identical dipeptides were also found, suggesting sequence similarity between the paramyosins. VY, a known anti-ACE peptide [23], was found in seafood paramyosins of five species (i.e., SC, WS, JA, MM, and PO). On the other hand, SL, a known anti-DPP-IV peptide [24], was found in seafood paramyosins of seven species, except for SC. Meanwhile, identical sets of anti-ACE and anti-DPP-IV peptides were found for both CO-X1 and CO-X2 as well as for both MM and PO (Table 2). Interestingly, some dipeptides in Table 2 (i.e., AY, IL, TF, VF, and VY) are bi-functional peptides. For instance, VY derived from five species (SC, WS, JA, MM and PO) has been reported to have both anti-ACE [23] and anti-DPP-IV [25] activity. One peptide sequence having bifunctional anti-ACE/anti-DPP-IV activities (VF) was released by HS paramyosin, whereas three bifunctional peptide sequences were found for WS (IL, TF, and VY) and JA (AY, IL, and VY). Taken together, when the number of peptides and bifunctionality are considered, the WS paramyosin was the most outstanding. It liberated three anti-ACE and 12 anti-DPP-IV peptides predicted as high-GI-absorbable, non-allergenic and non-toxic, among which three were bifunctional anti-ACE/anti-DPP-IV peptides. It should be noted that although other seafood proteins may also release similar high-GI-absorbable, non-allergenic and non-toxic anti-ACE and anti-DPP-IV peptides as paramyosins following GI digestions, we hypothesize based on our in silico scientific evidence that any anti-ACE and anti-DPP-IV effects of consumed seafoods could be attributed, at least partly, to paramyosins.

### 2.5. Predicting Anti-ACE and Anti-DPP-IV Peptides with SwissTargetPrediction

To further explore the possible presence of paramyosin-derived peptides which are novel, or not documented in the BIOPEP-UWM database, we adopted a target-fishing strategy. All paramyosin-derived peptides (2587) not recognized as anti-ACE and anti-DPP-IV peptides by BIOPEP-UWM were first screened for high GI absorption, non-allergenicity, and non-toxicity. This reduced the number of peptides to 64. Altogether, they can be narrowed down to four unique sequences: AK, DL, GIL and IAL (Table 3). Among the nine paramyosins investigated, the paramyosin of JA was the one from which these four high-GI-absorption, non-allergenic, and non-toxic peptides without any known anti-ACE and anti-DPP-IV activities can be found. On the other hand, AK can be consistently found in all nine paramyosins. DL can be found from all paramyosins, except for that of SC. This set of four peptides were analyzed with SwissTargetPrediction tool to predict potential anti-ACE and anti-DPP-IV peptides, based on their structural similarity to drug or compounds known to be ligands to ACE and to DPP-IV. This step led to the discovery of three putative anti-ACE peptides (GIL, DL, AK) and one putative anti-DPP-IV peptide (IAL) (Table 4).

### 2.6. Molecular Docking

In order to clarify the mechanisms of interactions between the known/predicted anti-ACE/anti-DPP-IV peptides and their target enzymes, we analyzed them by performing molecular docking. Overall, the 26 known anti-ACE peptides liberated from the paramyosins through in silico GI digestion (Table 2) can be narrowed down to eight unique sequences of anti-ACE peptides: AY, CF, EF, GM, IL, TF, VF, and VY. While the anti-ACE activity of the eight peptides were previously demonstrated, their mechanisms of inhibition have not been elucidated for all of them. Molecular docking represents a fast and economical in silico tools to clarify the potential mechanisms of action of the eight peptides side-by-side in the same study. On the other hand, comparison of the ACE-binding modes of the predicted anti-ACE peptides with those of the eight known anti-ACE peptides may also provide hints on the former’s anti-ACE potential. As shown in Table 5, the eight known anti-ACE peptides ranged between −112.800 (VY) to −75.728 (GM) in their docking scores. These scores are clearly inferior to the score of bradykinin potentiating peptide b (BPPb) (−376.180), which is the co-crystallized inhibitor in the human ACE crystal 4APJ. Underlying these weaker scores in the eight anti-ACE peptides could be their fewer interactions with ACE, in contrast with those formed between BPPb and ACE. Our results suggest that even at binding stability weaker than of BPPb, it is still possible for a peptide to be an effective ACE inhibitor. Based on comparison with the known anti-ACE peptides, it could be deduced that among the three predicted peptides, GIL was likely a potential anti-ACE peptide. This is due to the fact that it could bind to ACE with the strongest binding stability, which also falls within the score range exhibited by the eight known anti-ACE peptides. 

The active site of the ACE enzyme includes the inhibitor binding site (His383, His387, and Glu411), the S1 pocket (Ala354, Glu384, and Tyr523), the S2 pocket (Gln281, His353, Lys511, His513, and Tyr520), and S1′ (Glu162). An inhibitor that binds to ACE through other than the aforementioned active site is a non-competitive inhibitor [26]. Thus, our results in Table 5 implies that the eight known anti-ACE peptides derived from seafood paramyosins are mostly non-competitive ACE inhibitors (i.e., AY, CF, EF, IL, TF, VF, and VY). The role of VY as a non-competitive inhibitor of ACE was reported [27]. Our findings imply that the putatively high-GI-absorption, non-allergenic, and non-toxic anti-ACE peptides in all paramyosin sources, except SC, are all putatively non-competitive ACE inhibitors. By contrast, a combination of competitive (GM) and non-competitive (EF, IL, and VY) peptides could be derived from SC. Among the three anti-ACE peptides predicted by SwissTargetPrediction tool, two (GIL and DL) are possible non-competitive ACE inhibitors. Taken together, this prevalence of possible non-competitive ACE inhibitors is not unusual. In our previous in silico investigation of anti-ACE peptides from calpain 2-digested silkworm cocoon proteins, all four shortlisted dipeptides (AF, IL, PG and AG) were also deduced to be non-competitive ACE inhibitors [28].

The 53 known anti-DPP-IV peptides generated from the paramyosins through in silico GI digestion (Table 2) can be consolidated into eight unique sequences: AY, IL, SL, TF, TY, VF, VL, and VY. As shown in Table 4, IAL was the only putative anti-DPP-IV peptide predicted by SwissTargetPrediction. As presented in Table 6, the docking scores of TF, TY, VY and VF (−134.788 to −122.342) are clearly superior to the score of diprotin A (−115.228), which is the co-crystallized peptide inhibitor in the human DPP-IV crystal 1WCY. The stronger scores of the four anti-DPP-IV peptides may be partly accounted by their more frequent interactions with the active site residues of DPP-IV, in comparison with diprotin A. The four dipeptides formed 9–12 hydrophobic interactions with the residues in the DPP-IV active site, whereas diprotin A formed only seven. The active site of DPP-IV consists of a catalytic triad (Ser630, Asn710, and His740), a hydrophobic S1 pocket (Tyr631, Val656, Trp659, Tyr662, Tyr666, Val711), and a S2 pocket (Arg125, Glu205, Glu206, Ser209, Phe357, Arg358) [29]. Our molecular docking revealed that the eight known and one predicted anti-DPP-IV peptides could bind to at least one residue (Ser630) of the catalytic triad through hydrophobic interactions (Table 6). TF, TY, and AY were predicted to engage all three residues in the catalytic triad. Five known (TF, TY, AY, and IL) and one predicted (IAL) anti-DPP-IV peptides was also predicted to interact with His740 by hydrogen bonds. By contrast, diprotin A only bound to Ser630 of the DPP-IV catalytic triad via hydrophobic interaction (Table 6). Based on their modes of binding to the active site of DPP-IV, the paramyosin-derived peptides listed in Table 6 are all potentially competitive inhibitors. Our interpretation agrees with a previous report of SL being a competitive inhibitor of DPP-IV [30].

### 2.7. Molecular Dynamics

To further dissect the dynamics of interactions between the aforementioned anti-ACE and anti-DPP-IV peptides and their respective target proteins, we have performed 25 ns molecular dynamics (MD) simulations on selected anti-ACE and anti-DPP-IV peptides from both BIOPEP-UWM and SwissTargetPrediction results. Structural parameters RMSD, Radius of gyration (Rg), intermolecular H-bonds and protein-ligand distance were examined to determine the stability, dynamical behavior, and the compactness of the protein-ligand complexes.

#### 2.7.1. Root Mean Square Deviation

**Figure 2:**
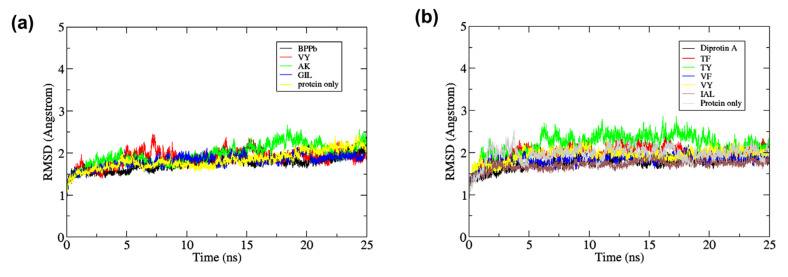
All-atom root mean square deviation (RMSD) of (**a**) Free ACE and ACE-peptide complexes (**b**) Free DPP-IV and DPP-IV-peptide complexes during 25 ns of the molecular dynamics simulation.

The root mean square deviation (RMSD) of the all-atom protein structure and the peptide ligand was employed to analyze each protein and ligand stability in complex. The mean RMSD value of protein-ligand that is lower than of the BPPb for ACE, and diprotin A for DPP-IV indicates a stable complex formation [31]. For ACE inhibitory peptide, VY and AK from BIOPEP-UWM and GIL from SwissTargetPrediction were subjected to 25 ns MD. The mean RMSD value of ACE complexed with BPPb was 1.75 ± 0.16 Å, while for VY, AK and GIL were 1.88 ± 0.19 Å, 1.96 ± 0.23 Å, and 1.81 ± 0.15 Å, respectively. While the mean RMSD value of complexed-ACE for the selected peptides were slightly higher than the BPPb, Figure 2a exhibit the overall stability of the RMSD for the whole 25 ns duration. The ACE-VY complex RMSD value was slightly fluctuated early at 7 ns, while for ACE-AK complex the value increased during 16–20 ns. The mean RMSD of free ACE protein at 1.88 ± 0.20 Å was higher than of the BPPb, VY and GIL complexes, and its graph shows gradual increase during the 25 ns course (Figure 2a). The low RMSD of protein-ligand complex in comparison with free protein also indicates a stable protein-ligand complex formation [32]. 

Similarly, the RMSD analysis for anti DPP-IV and peptide complexes was also conducted. Here, the inhibitory peptide candidates TF, TY, VF, VY, and IAL which form complexes with DPP-IV, and DPP-IV- diprotin A complex were subjected to 25 ns MD simulation. The all-atom protein mean RMSD value for DPP-IV-diprotin A complex was lower (1.76 ± 0.15 Å) compared to all dipeptides subjected in the study (TF = 2.00 ± 0.15 Å, TY = 2.20 ± 0.25 Å, VF = 1.82 ± 0.12 Å, VY = 1.93 ± 0.15 Å). However, the tripeptide IAL (1.71 ± 0.11 Å) showed a considerably low mean RMSD value against Diprotin A, which suggested a stable protein-ligand binding [33]. In comparison, the mean RMSD of free DPP-IV was higher at 1.96 ± 0.19 Å. The all-atom protein RMSD plotted in Figure 2b shows DPP-IV that formed complexes with each diprotin A and IAL were stable during 25 ns compared to free DPP-IV which was highly fluctuated, especially at the few first ns of the simulation. In addition, DPP-IV-TY complex gave a high all-atom protein RMSD fluctuation during 25 ns, even though it had a lower docking score predicted by the BIOPEP-UWM server compared to diprotin A. 

**Figure 3:**
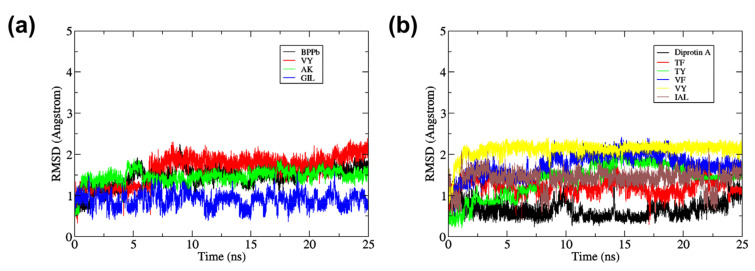
All-atom root mean square deviation (RMSD) of (**a**) ACE-docked peptide complexes (**b**) DPP-IV-docked peptide complexes during 25 ns of the molecular dynamics simulation.

**Figure 4:**
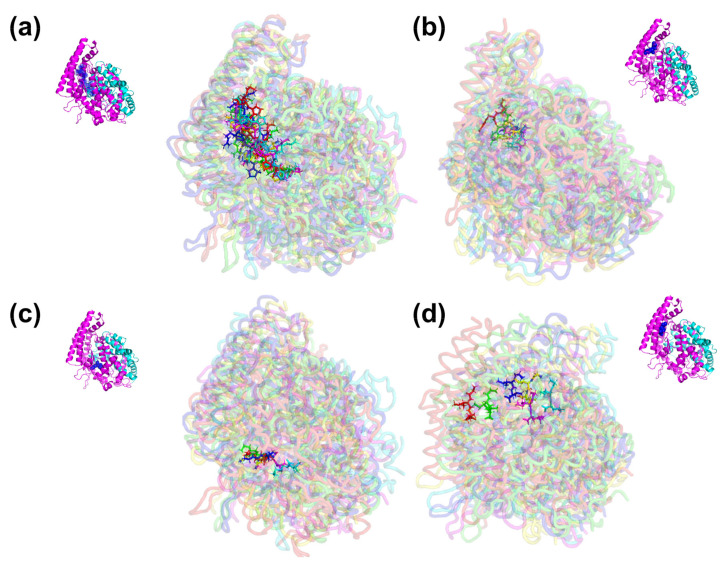
Snapshot of the superimposed structures of ACE in complex with peptide (**a**) BPPb, (**b**) VY, (**c**) AK and (**d**) GIL. Structures were obtained from the trajectory file in the interval of 5 ns for 25 ns MD.

To ensure the binding stability of inhibitory peptides in the active site of ACE and DPP-IV, the ligand positional all-atom RMSD was also calculated. This is also important as from the docking result, the screened peptides were suggested to be the non-competitive inhibitors as it binds to the site other than the inhibitor binding site, the S1 pocket, the S2 pocket and S1′. Figure 3a shows that each BPPb, VY, and AK gave a stable all-atom ligand RMSD when complexed with ACE protein with the mean RMSD value of 1.45 ± 0.24 Å, 1.65 ± 0.33 Å and 1.46 ± 0.17 Å, respectively. In comparison, GIL mean RMSD value was a magnitude lower at 0.89 ± 0.16 Å, although the plot shows that its RMSD fluctuated for the whole 25 ns. Therefore, five snapshots of ACE-peptide complexes were downloaded in the interval of 5 ns during the entire simulation, as in Figure 4a–d. It was observed that docked GIL had moved considerably at the active site during the intervals, while BPPb, VY and AK remained firmly bound at the active site of ACE. BPPb occupied the most active site regions due to its large decapeptide structure which gave a spatial space around the binding pockets. While VY and GIL ligand shared the similar binding region of alpha-helices domain where Glu123 and residues Tyr51, Trp59, Tyr62, and Ala63 were located, AK tends to bind on the different region where residues Glu162, Cys352 and Lys511 were located and highly interacted with the N-terminal and O-terminal of the peptide.

**Figure 5:**
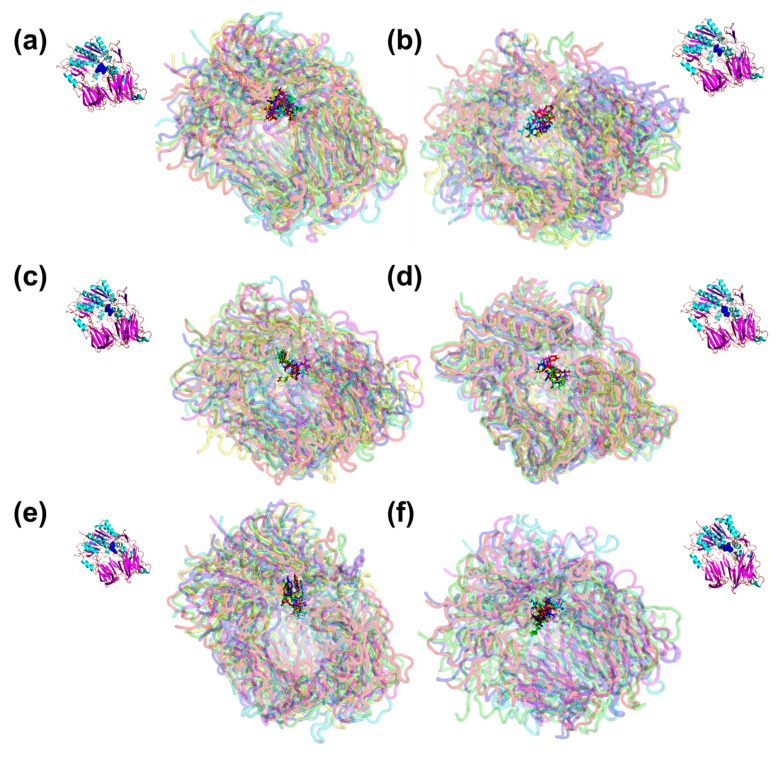
Snapshot of the superimposed structures of DPP-IV in complex with peptide (**a**) Diprotin A, (**b**) TF, (**c**) TY, (**d**) VF, (**e**) VY and (**f**) IAL. Structures were obtained from the trajectory file in the interval of 5 ns for 25 ns.

As the GIL inhibitory peptide predicted from SwissTargetPrediction gave a lower ligand RMSD compared to the positive control peptide (BPPb) as the ACE inhibitor candidate, the same cannot be said for the peptide ligand against DPP-IV. DPP-IV peptide inhibitor diprotin A gave a relatively low ligand RMSD value as observed in the plot Figure 3b with the mean RMSD of 0.64 ± 0.19 Å, compared to the dipeptide inhibitors suggested by BIOPEP-UWM which gave a higher mean RMSD values (TF = 1.22 ± 0.17 Å, TY = 1.39 ± 0.41 Å, VF = 1.74 ± 0.28 Å and VY = 2.11 ± 0.18 Å). However, IAL peptide obtained from SwissTargetPrediction gave a considerably low ligand RMSD with the mean value of 1.41 ± 0.22 Å when compared to the dipeptides obtained from BIOPEP-UWM. The snapshots in Figure 5a–f shows that the docked region of diprotin A and other inhibitor peptides candidates shared the similar binding residues of Arg125, Glu205 and the pockets residues around Glu206, Tyr547, Trp629, Ser630, and His740. 

#### 2.7.2. Radius of Gyration

Radius of gyration (Rg) measures the compactness of a protein which allows the understanding of protein folding properties [34]. While the Gromacs tool ‘gmx_gyrate’ is applicable to compute the protein radius of gyration, the equation can be written as below;

(1) $$R_{g}^{2}=\frac{1}{M}\overset{N}{\underset{i=1}{\sum }}m_{i}{\left(r_{i}-R\right)}^{2}$$

where $M=\overset{N}{\underset{i=1}{\sum }}m_{i}$ is the total mass, and $R={\;N}^{-1}\sum_{i=1}^{N}r_{i}$ is the center of mass of the protein consisting of N atoms. A small Rg values shows that the protein is in a tight packing with a relatively steady value of Rg, while high Rg values indicate a floppy packing of protein with lack of compactness. In addition, a stable Rg value of protein-ligand complex during the time frame indicates that the ligand holds the folding behavior of protein whilst high Rg fluctuations might denotes the protein-ligand folding instability over time [35].

**Figure 6:**
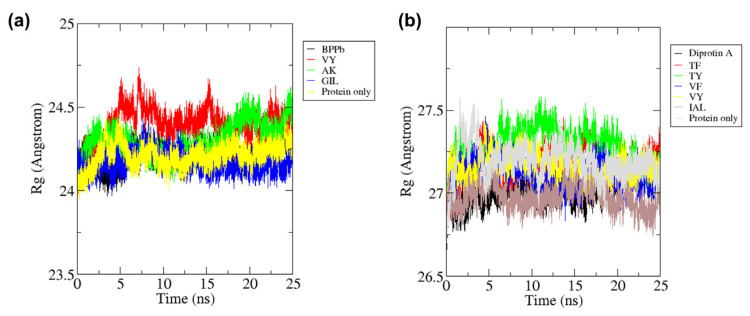
All-atom radius of gyration of (**a**) ACE and (**b**) DPP-IV as free proteins and forming complexes with the inhibitor peptides during 25 ns of the molecular dynamics simulation period.

The free ACE protein (mean Rg = 2.42 nm) was shown to have similar compactness with the ACE-BPPb complex (mean Rg = 2.42 nm) and ACE-GIL (mean Rg = 2.42 nm), while the Rg value was slightly higher against two dipeptides VY (mean Rg = 2.44 nm) and AK (mean Rg = 2.43 nm) after 25 ns. The gyration of ACE-VY and ACE-AK complexes were observed to be fluctuated for the whole 25 ns compared to free ACE, as observed in Figure 6a. The gyration of ACE-GIL complex was shown to be more stable during the period. In comparison, free DPP-IV protein and each of its peptide ligand complex are slightly less compact, with the free DPP-IV Rg mean value of 2.72 nm, followed by diprotin A (mean Rg = 2.70 nm), TF (mean Rg = 2.72 nm), TY (mean Rg = 2.72 nm), VF (mean Rg = 2.71 nm), VY (mean Rg = 2.72 nm) and IAL (mean Rg = 2.70 nm), as observed in Figure 6b. The high fluctuations of free DPP-IV and DPP-IV peptide complexes was mainly caused by higher number of residues in the protein and wider space for protein-ligand spatial interaction in the protein active site [36]. The Rg kept constant with no abrupt fluctuations through the time in all of these complexes, indicating that VY, AK, and GIL maintain the folding behavior as similarly as ACE-BPPb complex, while TF, TY, VF, VY, and IAL maintains DPP-IV folding as similarly from the complex formation with diprotin A (Figure 6). 

#### 2.7.3. Hydrogen Bonds and Protein-Ligand Distance

Hydrogen bonds (H-bonds) are non-covalent bonds that provide most of the directional interactions underneath the formation of secondary and tertiary structure protein motifs where it satisfies the hydrogen-bonding potential between carbonyl oxygen and amide nitrogen in the hydrophobic core of protein [37]. Close proximity of the polar atoms in protein and its ligand, with acceptor-donor distance between 2.0 and 2.5 Å and its geometric angle of less than 120° also provides a directionality and specificity of the H-bond interaction. In addition, it also explains the binding affinity of a ligand towards the protein target in the molecular dynamics simulation. Therefore, a higher number of intermolecular H-bonds can be translated to stronger interactions between the complex and smaller protein-ligand intermolecular distance [38].

**Figure 7:**
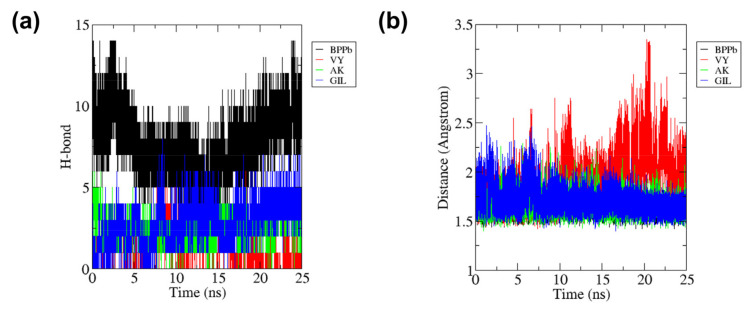
(**a**) Total number of hydrogen bonds interactions between ACE and each peptide and (**b**) intermolecular distance of ACE with peptides during 25 ns of the molecular dynamics simulation period.

Figure 7a shows that the ACE protein complexed with BPPb provides the highest number of intermolecular H-bonds with mean seven bonds, while AK and GIL provide the mean H-bonds of three bonds followed by VY with only one bond during 25 ns simulation. Number of H-bonds formed by ACE-BPPb complex was high while H-bonds between ACE-GIL complex seems to increase within 25 ns. Figure 7b shows that these were translated to the protein-ligand intermolecular distance where ACE-BPPb has the smallest distance of 2.0 Å, ACE-VY complex distance slowly increased to more than 3.0 Å after 15 ns and was highly fluctuated. On the other hand, ACE-GIL complex tends to stabilize after 10 ns and the distance decrease to less than 2.0 Å until the end of the duration. 

**Figure 8:**
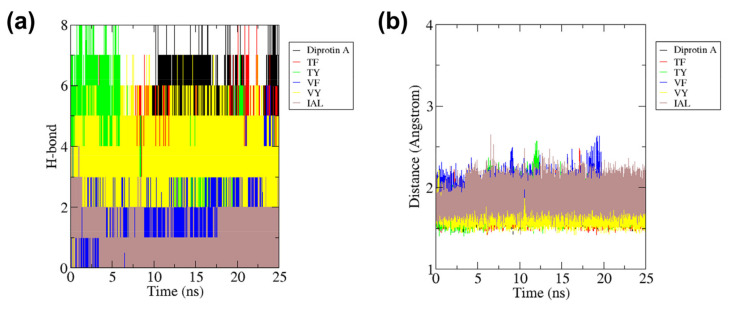
(**a**) Total number of hydrogen bonds interactions between DPP-IV and each peptide and (**b**) intermolecular distance of DPP-IV with peptides during 25 ns of the molecular dynamics simulation period.

Similarly, for anti-DPP-IV peptides, DPP-IV-diprotin A complex formed the most H-bonds with mean four bonds, followed by TF, TY and VY with the mean three bonds each (Figure 8a). IAL and DPP-IV complex has the lowest intermolecular H-bond with only one bond, where the amide group on the N-terminal of IAL bonded with either residues Glu205 or Glu206 of the DPP-IV during the simulation (not shown). Due to the pocketed position of the peptide ligand which is in the close proximity with the surrounded alpha-helices domains, the peptides were also supported by strong hydrophobic interactions and tend to stabilize. This also contributed to the intermolecular distance of protein-ligand for each complex were low and less than 2.5 Å each (Figure 8b). 

### 2.8. SwissADME Analysis

SwissADME is a free web tool used in some in silico studies to assess the physicochemical properties, pharmacokinetics and drug-likeness of bioactive peptides [39,40]. Based on the predicted information, it may be possible to compare the peptides under investigation with approved peptide drugs [39]. Table 7 shows the physicochemical properties of the 15 unique sequences of high-GI-absorption, non-allergenic, non-toxic anti-ACE and/or anti-DPP-IV peptides derived from the seafood paramyosins. The physicochemical properties of the 15 peptides are generally comparable to those of Captopril and Anagliptin. Captopril is an ACE inhibitor used as an oral antihypertension drug [8]. Anagliptin is an anti-DPP-IV agent used as an oral antidiabetic drug [11]. The 15 peptides range between 217 g/mol (AK) and 315 g/mol (IAL) in molecular weights, not remarkably different from the two small-molecule drugs Captopril and Anagliptin. Meanwhile, the majority of the United States Food and Drug Administration (FDA)-approved, orally available peptide drugs have the following characteristics: fraction Csp3 up to 0.55; rotatable bonds (RB) up to 20; number of H-bond acceptors (HBA) up to 50; number of H-bond donors (HBD) up to 25; TPSA up to 400 Å^2^; and lipophilicity between −5 and 8 [41]. It is clear from Table 7 that the RB, HBA, HBD, TPSA, and lipophilicity values of all of the 15 peptides are comparable to those found in orally available peptide drugs. However, only seven peptides (AY, CF, EF, TF, TY, VF, and VY) have fraction Csp3 values smaller than 0.55. Taken together, based on the physicochemical descriptors in Table 7, only the seven aforementioned dipeptides resemble FDA-approved, orally available peptide drugs. 

Table 8 shows the predicted pharmacokinetic properties, drug-likeness and lead-likeness of the 15 selected anti-ACE/anti-DPP-IV peptides shortlisted from seafood paramyosins in this study. All 15 peptides were predicted to be non-substrates of P-glycoprotein (P-gp). P-gp is one of the drug transporters that regulate the update and efflux of drugs in the body. It is known to reduce the oral bioavailability of its substrates. Furthermore, P-gp substrates may potentially act as inducers or inhibitors of P-gp. This could enhance the risk of drug-drug interactions, particularly involving drugs acting on P-gp [42]. That the 15 peptides were predicted as non-P-gp-substrates is therefore desirable, implying that their oral bioavailability would not be compromised by P-gp, neither would the peptides bring about risks of drug-drug interactions. On the other hand, the 15 peptides were predicted as non-inhibitors of all five cytochrome P450 (CYP) isozymes (CYP1A2, CYP2C19, CYP2C9, CYP2D6 and CYP3A4) (Table 8). The aforementioned enzymes play an important role in Phase I biotransformation. Inactivation of the CYP enzymes by a drug or other molecules may cause bioaccumulation and, consequently, toxicity [43]. The predicted non-inhibition of the CYP isozymes by the 15 peptides is therefore consistent with their predicted non-toxicity by Toxinpred in this study. The 15 peptides are comparable to the anti-ACE antihypertension drug Captopril based on the peptides’ status as non-P-gp substrates and non-inhibitors of CYP isozymes. Theoretically, the 15 peptides are also less likely to raise risk of drug-drug interactions relative to the anti-DPP-IV antidiabetic drug Anagliptin, a P-gp substrate. In terms of drug-likeness, all 15 paramyosin-derived peptides were predicted to comply with the Lipinski’s rule-of-five, with zero violations each. The Abbot bioavailability score estimates the probability that a compound has at least 10% oral bioavailability in the rat or measurable Caco-2 permeability [44]. Similar scores were predicted among the 15 peptides and the two oral drugs we used for comparison. The observation suggests that all 15 peptides can be considered as oral drug candidates [45] that can be further tested for their in vivo effects. In the context of drug development, six of the 15 paramyosin-derived peptides stood out in terms of lead-likeness: AY, CF, TF, TY, VF, and VY. These six dipeptides are also among the seven peptides resembling FDA-approved, orally available peptide drugs based on the physicochemical descriptors in Table 7. Theoretically, the six dipeptides are considered suitable to be subjected to further chemical modifications for lead optimization [45]. 

**Figure 9:**
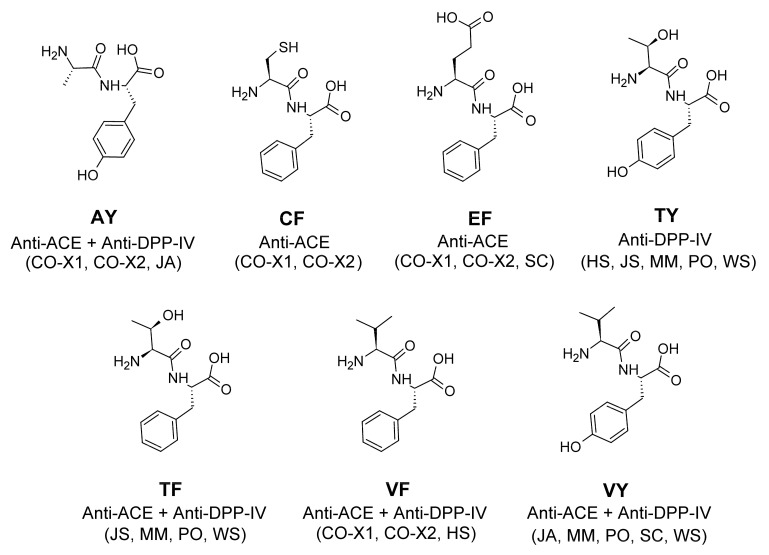
Seven putative drug-like peptides derived from seafood paramyosins.

In summary, based on our SwissADME analysis, we found seven putative drug-like peptides (AY, CF, EF, TF, TY, VF, and VY) resembling FDA-approved oral peptide drugs (Figure 9). Among these seven, AY, CF, EF, TF, VF, and VY were demonstrated anti-ACE peptides, whereas AY, TF, TY, VF, and VY were demonstrated anti-DPP-IV peptides. These two sets could be consolidated into four bifunctional anti-ACE/anti-DPP-IV peptides: AY, TF, VF and VY. Notably, all seven peptides have an aromatic residue (F or Y) in their C-termini. Overall, our results suggest that the nine paramyosins investigated can serve as sources of bioavailable, safe, single/dual-function anti-ACE and anti-DPP-IV peptides upon oral ingestion and GI digestion. Considering the in silico/theoretical nature of this study, the actual pool of anti-ACE and anti-DPP-IV peptides liberated from the paramyosins as well as the activity of the three putative anti-ACE peptides (GIL, DL, AK) and one putative anti-DPP-IV peptide (IAL) must be validated in future wet lab experiments. Notably, this virtual screening study has pinpointed promising candidates that can be prioritized in future investigations. Among the nine paramyosins investigated, the two paramyosins isoforms of the common octopus appear to be the most frequent sources of drug-like peptides exhibiting either only anti-ACE activity (CF and EF) or anti-ACE + anti-DPP-IV activities (AY and VF) (Figure 9). Thus, for future discovery of food-derived nutraceuticals or drug candidates targeting hypertension and/or diabetes, the common octopus paramyosins represent a desirable raw material. 

## 3. Materials and Methods

### 3.1. Paramyosin Protein Sequences

**Figure 10:**
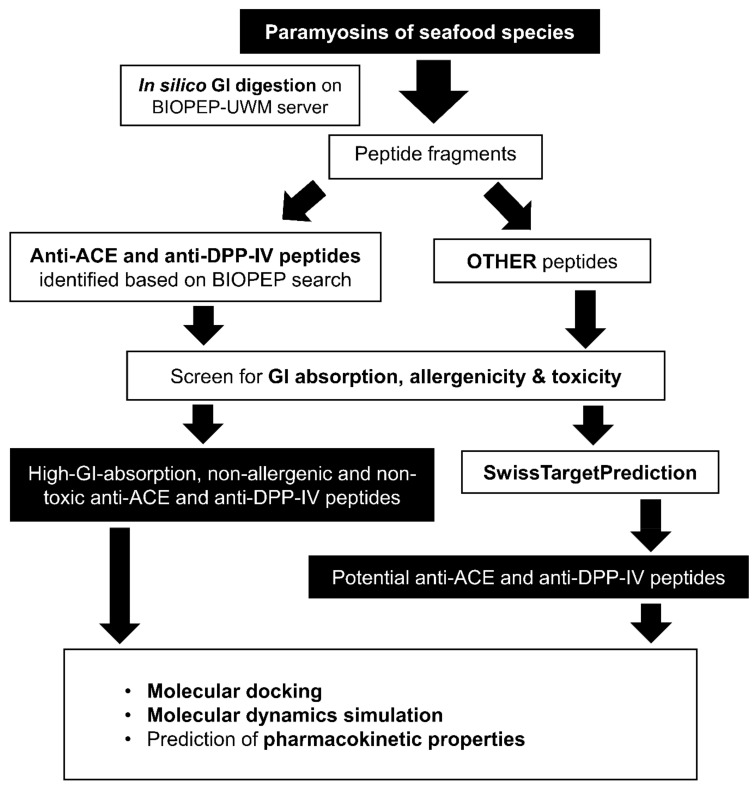
An overview of the study.

The protein sequences of paramyosins of eight seafood species were retrieved from the UniProt Knowledgebase (UniProtKB) (https://www.uniprot.org/, accessed on 28 August 2021) [46] in the FASTA format. The eight species were the common octopus (*Octopus vulgaris*), Humboldt squid (*Dosidicus gigas*), Japanese abalone (*Haliotis discus hannai*), Japanese scallop (*Mizuhopecten yessoensis*), Mediterranean mussel (*Mytilus galloprovincialis*), Pacific oyster (*Crassostrea gigas*), sea cucumber (*Stichopus japonicus*), and Whiteleg shrimp (*Penaeus vannamei*). The number of residues and molecular mass of each paramyosin protein were recorded. In this study, the protein sequences retrieved from UniProtKB were used in in silico GI digestion. Fragments liberated from the digestion were used in in silico screening for high-GI-absorption, non-allergenic, and non-toxic anti-ACE and anti-DPP-IV peptides, as depicted in Figure 10.

### 3.2. In Silico GI Digestion of Paramyosins

The paramyosin sequences were subjected to in silico GI digestion on the BIOPEP-UWM server (https://biochemia.uwm.edu.pl/en/start/, accessed on 8 September 2021) [21] using the “enzyme(s) action” tool. Chymotrypsin A (EC 3.4.21.1), trypsin (EC 3.4.21.4), and pepsin (pH 1.3) (EC 3.4.23.1) were used for in silico GI digestion as previously reported [20]. The peptide fragments released from each protein were recorded and divided into separate groups: two residues, three residues, four residues, and >four residues. Peptides with previously demonstrated anti-ACE and anti-DPP-IV activities were identified by using the “Search for active fragments” tool in BIOPEP-UWM. 

### 3.3. Prediction of GI Absorption, Allergenicity, and Toxicity of Peptides

Peptides released from the in silico GI digestion of paramyosins were screened for GI absorption in SwissADME (http://www.swissadme.ch/, accessed on 17 September 2021) [45]. The conversion of peptide sequences into the Simplified Molecular Input Line Entry System (SMILES) format was conducted with the “SMILES” tool of BIOPEP-UWM; the SMILES strings were then submitted to SwissADME as input for analysis. Peptide allergenicity was screened with AllerTOP v.2.0 (https://www.ddgpharmfac.net/AllerTOP/, accessed on 17 September 2021) [47]. Toxicity was screened with ToxinPred (https://webs.iiitd.edu.in/raghava/toxinpred/index.html, accessed on 17 September 2021) [48].

### 3.4. Ligand-Based In Silico Target Fishing

High-GI-absorption, non-allergenic, and non-toxic peptides not indicated as having anti-ACE and anti-DPP-IV activities based on BIOPEP-UWM search were further analysed with SwissTargetPrediction (http://www.swisstargetprediction.ch/, accessed on 20 September 2021). SwissTargetPrediction is a free, web-based tool that can be used to predict putative human protein targets of any small molecules. Through reverse screening, target prediction is accomplished by matching the structures of query compounds to similar two-dimensional (2D) and three-dimensional (3D) structures of compounds experimentally active on human protein targets [49]. For this analysis, the “Select a species” was set to “*Homo sapiens*”. Peptide sequences in the SMILES format were generated with BIOPEP-UWM as described in Section 3.3 (accessed on 20 September 2021). Output of prediction was ranked based on the “Known actives (3D/2D)” parameter. Peptides whose top predicted target was ACE or DPP-IV were recorded, along with the probability of the prediction.

### 3.5. Molecular Docking Analysis

The docking of peptides onto ACE and DPP-IV was accomplished with HPEPDOCK (http://huanglab.phys.hust.edu.cn/hpepdock/, accessed on 21 December 2021) [50]. The crystal structures of ACE and DPP-IV were downloaded from RCSB Protein Data Bank (https://www.rcsb.org/, accessed on 21 December 2021) [51]. The crystal of the human ACE was complexed with bradykinin potentiating peptide b (BPPb)(PDB ID: 4APJ) [52], whereas the human DPP-IV was complexed with diprotin A (PDB ID: 1WCY) [53]. Upon removal of the bound ligands, the receptors (ACE and DPP-IV) were subjected to energy minimization in the Swiss-PdbViewer 4.0 software [54] prior to docking with HPEPDOCK. To ensure suitability of docking procedure, redocking of co-crystalized ligands (BPPb and diprotin A) to their respective crystals were performed with HPEPDOCK. Peptides were submitted to HPEPDOCK in the PDB format. The 3D structures of peptides were retrieved from Mendeley Data. (https://data.mendeley.com/datasets/z8zh5rpthg/1, accessed on 26 September 2021) [55] and converted into the PDB format by using BIOVIA Discovery Studio Visualizer (BIOVIA, Dassault Systèmes, BIOVIA Discovery Studio Visualizer, Version 20.1.0.192, San Diego: Dassault Systèmes, 2020). The top (most negative) docking score for each peptide-ACE or peptide-DPP-IV docking, as reported by HPEPDOCK, was recorded. BIOVIA Discovery Studio Visualizer was used for the visualization of the 3D structures of the docked models generated by HPEPDOCK. LigPlot+ v.2.2 was used for the 2D visualization and analysis of intermolecular interactions between a peptide and the target proteins [56,57].

### 3.6. Molecular Dynamics Analysis 

Molecular dynamics (MD) was performed in GROMACS 2020 using AMBER99SB-ILDN force field. MD simulation was performed on free proteins (ACE and DPP-IV), docked peptide-ACE and peptide-DPP-IV complexes. Similarly, MD of ACE- BPPb complex and DPP-IV-diprotin A complex were also performed as control. In the MD, each complex was solvated in a cubic box with a distance of 1.2 nm between the complex and each side of the solvated box, and sodium and chloride ions were added to neutralize the total charge of the system [58]. The complex was then energy-minimized using the steepest descent algorithm. The simulation conditions were set at room temperature (300 K) and atmospheric pressure (1 bar) to mimic the general experiment conditions. The fully temperature and pressure equilibrated system was treated as the minimization step for the complex and used as the initial configuration for the MD production dynamic analysis. All simulations were conducted for 25 ns using a 2 fs time step. The results then were analyzed using common GROMACS functions RMSD and RMSF, while the formation of the intermolecular hydrogen bonds in the complex were analyzed using ‘gmx_hbond’ function. Radius of gyration for free and protein-ligand complexes were also analyzed. The intermolecular distance between each ACE and DPP-IV and their peptide ligand was measured using the ‘gmx_pairdist’ function.

### 3.7. Prediction of Physicochemical and Pharmacokinetic Properties

The physicochemical and pharmacokinetic properties of selected peptides were assessed using SwissADME (http://www.swissadme.ch/, accessed on 27 January 2022) [45]. Peptide sequences in the SMILES format were generated as described in Section 3.3 (access date: 27 January 2022). Physicochemical properties, as well as other predicted information concerning the pharmacokinetics, drug-likeness, and lead-likeness of the peptides was recorded. The 2D structures of selected peptides were drawn by using the ACD/ChemSketch freeware (ACD/ChemSketch, version 2019.2.1, Advanced Chemistry Development, Inc., Toronto, ON, Canada, www.acdlabs.com, 2019).

## Figures and Tables

**Table 1:** Length and molecular masses of paramyosin proteins of eight seafood species.

Seafood	Accession Number	Number of Residues	Mass (Da)
Common octopus (CO-X1)	A0A6P7TIV8 (isoform X1)	523	59,847
Common octopus (CO-X2)	A0A7E6FQ28 (isoform X2)	516	59,026
Humboldt squid (HS)	A0A1Y1DCG9	880	102,476
Japanese abalone (JA)	A0A286QYA2	860	99,648
Japanese scallop (JS)	A0A210R0B2	934	107,548
Mediterranean mussel (MM)	O96064	864	99,573
Pacific oyster (PO)	K1QTC1	851	97,876
Sea cucumber (SC)	A0A2G8LGY5	727	83,851
Whiteleg shrimp (WS)	A0A3R7QCP1	828	96,537

**Table 2:** The numbers of high-GI-absorption, non-allergenic, and non-toxic peptides with known anti-ACE and anti-DPP-IV activities.

Seafood	Anti-ACE Peptides		Anti-DPP-IV Peptides	
	Number	Unique Sequences ^a^	Number	Unique Sequences ^a^
CO-X1	5	AY, CF, EF, VF	6	AY, SL, VF, VL
SC	5	EF, GM, IL, VY	4	IL, VL, VY
CO-X2	4	AY, CF, EF, VF	6	AY, SL, VF, VL
WS	3	IL, TF, VY	12	IL, SL, TF, TY, VL, VY
JA	3	AY, IL, VY	5	AY, IL, SL, VY
MM	2	TF, VY	5	SL, TF, TY, VY
PO	2	TF, VY	5	SL, TF, TY, VY
JS	1	TF	6	SL, TF, TY, VL
HS	1	VF	4	SL, TY, VF
Total	26		53	

^a^ Bifunctional dipeptides with both anti-ACE and anti-DPP-IV activities are underlined.

**Table 3:** The numbers of high-GI-absorption, non-allergenic, and non-toxic peptides without known anti-ACE and anti-DPP-IV activities.

Seafood	Number	Unique Sequences
JA	12	AK, DL, GIL, IAL
JS	10	AK, DL
PO	9	AK, DL
WS	8	AK, DL
MM	8	AK, DL
SC	6	AK, IAL
HS	5	AK, DL
CO-X1	3	AK, DL
CO-X2	3	AK, DL
Total	64	

**Table 4:** Peptide sequences having ACE or DPP-IV as potential target as predicted by SwissTargetPrediction.

Peptide	Potential Target	Probability	Known Actives (3D/2D)	ChEMBL ID of Known Active Compound with Top Similarity to Peptide */IC_50_
GIL	ACE	0.5345	167/189	CHEMBL128399/4200 nM
DL	ACE	0.0580	33/130	CHEMBL358439/2400 nM
AK	ACE	0.0524	2/183	CHEMBL430554/7 nM
IAL	DPP-IV	0.5776	167/362	CHEMBL214381/2530 nM

* Based on 3D structure comparison to known anti-ACE/anti-DPP-IV compounds stored in ChEMBLE database.

**Table 5:** Docking scores and intermolecular interactions between ACE and known/predicted anti-ACE peptides.

	Peptide	Docking Score	Interaction with ACE ^b,c^		
			Hydrogen Bond	Hydrophobic Interaction	Salt Bridge
	BPPb ^a^	−376.180	Lys118, Asp121, Gln281, Ala356(2), Tyr360, Glu403, Lys511, His513, Ser516, Ser517, Tyr520, Tyr523	Trp59, Ile88, Lys118, Asp121, Glu123, Gln281, His353, Ala354, Ser355, Ala356, Trp357, Tyr360, His387, Glu403, Glu411, Phe457, Lys511, Phe512, His513, Ser516, Ser517, Val518, Tyr520, Tyr523	Glu403
**Indicated by BIOPEP-UWM**	VY	−112.800	Glu123	Tyr51, Trp59, Tyr62, Ala63, Ile88, Lys118, Glu123, Tyr360	-
	CF	−108.762	Tyr62, Leu122, Glu123, Ala125	Trp59, Tyr62, Thr92, Glu123, Arg124, Ala125, Tyr360	-
	AY	−108.695	Glu123, Arg124, Tyr135, Asn211, Ser517	Glu123, Arg124, Tyr135, Leu139, Ile204, Ala207, Ala208, Ser219, Trp220, Ser517	-
	VF	−107.589	Glu123	Trp59, Tyr62, Ile88, Thr92, Leu122, Glu123, Arg124, Tyr360	-
	TF	−103.827	Tyr51, Glu123	Tyr51, Trp59, Ile88, His91, Thr92, Lys118, Asp121, Glu123	-
	EF	−103.021	Glu123, Arg124, Tyr135	Glu123, Arg124, Tyr135, Leu139, Ile204, Ala207, Ser219, Trp220, Ser517, Val518, Pro519, Arg522	Arg522(4)
	IL	−79.044	Tyr62, Asn85	Trp59, Tyr62, Asn85, Ile88, Ala89, Arg124, Leu132	-
	GM	−75.728	Tyr146, Phe512	Tyr146, Leu161, Glu162, Trp279, His353, Lys511, Phe512, His513	-
**Predicted by Swiss Target Prediction**	GIL	−103.475	Asp121, Glu123	Trp59, Tyr62, Ile88, Ala89, Thr92, Asp121, Leu122, Glu123, Arg124, Ala125	-
	AK	−64.629	Glu162, Lys511(2), His513	Tyr146, Leu161, Glu162, Trp279, Gln281, His353, Lys511, Phe512, His513	-
	DL	−60.501	Tyr62, Glu123, Arg124	Tyr62, Asn85, Ile88, Ala89, Glu123, Arg124	Arg124

^a^ Bradykinin potentiating peptide b, co-crystalized inhibitor of ACE in 4APJ crystal. ^b^ Residues in the active site of ACE are underlined. ^c^ The number in bracket indicates the number of hydrogen bonds or salt bridges formed with the same residues of ACE.

**Table 6:** Docking scores and intermolecular interactions between DPP-IV and known/predicted anti-DPP-IV peptides.

	Peptide	Docking Score	Interaction with DPP-IV ^b^	
			Hydrogen Bond ^c^	Hydrophobic Interaction
	Diprotin A ^a^	−115.228	Arg125(2), Glu205, Glu206(2), Tyr547, Tyr662	Arg125, Glu205, Glu206, Phe357, Tyr547, Ser630, Tyr631, Tyr662, Tyr666
**Indicated by BIOPEP-UWM**	TF	−134.788	Glu205(2), Glu206, Tyr662, His740	Arg125, Glu205, Glu206, Tyr547, Ser630, Tyr631, Val656, Trp659, Tyr662, Tyr666, Asn710, Val711, His740
	TY	−130.756	Glu205(2), Glu206, Tyr662, His740	Arg125, Glu205, Glu206, Tyr547, Ser630, Tyr631, Val656, Trp659, Tyr662, Tyr666, Asn710, Val711, His740
	VY	−125.108	Arg125, Glu205(2), Tyr547	Arg125, Glu205, Glu206, Ser209, Tyr547, Ser630, Tyr631, Val656, Trp659, Tyr662, Tyr666
	VF	−122.342	Glu205(2), Glu206	Arg125, Glu205, Glu206, Phe357, Tyr547, Ser630, Tyr631, Tyr662, Tyr666, Asn710
	AY	−114.150	Arg125, Tyr547, Ser630, His740	Arg125, Tyr547, Ser630, Tyr631, Val656, Trp659, Tyr662, Tyr666, Asn710, Val711, His740
	IL	−86.409	Glu205, Glu206, Tyr547, Ser630, His740	Glu206, Phe357, Tyr547, Ser630, Tyr631, Val656, Trp659, Tyr662, Tyr666, His740
	SL	−85.505	Glu205, Glu206(2), Tyr547, Tyr631, Tyr662(2)	Arg125, Glu205, Glu206, Tyr547, Ser630, Tyr631, Val656, Trp659, Tyr662, Tyr666, Val711
	VL	−84.356	Arg125, Glu205(3)	Arg125, Glu205, Glu206, Tyr547, Ser630, Tyr631, Tyr662, Tyr666, Arg669
**Predicted by Swiss Target Prediction**	IAL	−109.567	Arg125, Glu205, Glu206, Tyr547, Tyr662, His740	Arg125, Glu205, Glu206, Tyr547, Trp629, Ser630, Tyr662, Tyr666, Val711, His740

^a^ Bound ligand of DPP-IV in the crystal (PDB ID: 1WCY). ^b^ Residues in the active site of DPP-IV are underlined. ^c^ The number in the brackets indicates the number of hydrogen bonds formed with the same residues of DPP-IV.

**Table 7:** Physicochemical properties of peptides having known and potential anti-ACE/anti-DPP-IV activities, in comparison with Captopril (antihypertension drug) and Anagliptin (antidiabetic drug).

Peptide ^a^	MW (g/mol)	Fraction Csp3	RB	HBA	HBD	TPSA (Å^2^)	Lipophilicity (Consensus Log *P*_o/w_)
AK	217.27	0.78	8	5	4	118.44	−0.96
AY	252.27	0.33	6	5	4	112.65	−0.54
CF	268.33	0.33	7	4	3	131.22	−0.02
DL	246.26	0.70	8	6	4	129.72	−0.93
EF	294.30	0.36	9	6	4	129.72	−0.21
GIL	301.38	0.79	11	5	4	121.52	0.05
GM	206.26	0.71	7	4	3	117.72	−0.82
IAL	315.41	0.80	11	5	4	121.52	0.58
IL	244.33	0.83	8	4	3	92.42	0.49
SL	218.25	0.78	7	5	4	112.65	−0.80
TF	266.29	0.38	7	5	4	112.65	−0.52
TY	282.29	0.38	7	6	5	132.88	−0.97
VF	264.32	0.43	7	4	3	92.42	0.44
VL	230.30	0.82	7	4	3	92.42	0.26
VY	280.32	0.43	7	5	4	112.65	0.01
Captopril	217.29	0.78	4	3	1	96.41	0.62
Anagliptin	383.45	0.53	8	6	2	115.42	0.73

^a^ MW, molecular weight; fraction Csp3, the ratio of sp^3^ hybridized carbons over the total carbon count of the molecule; RB, number of rotatable bonds; HBA, number of H-bond acceptors; HBD, number of H-bond donors; TPSA, topological polar surface area.

**Table 8:** Pharmacokinetics, drug-likeness and lead-likeness of peptides having known and potential anti-ACE and anti-DPP-IV activities, in comparison with Captopril (antihypertension drug) and Anagliptin (antidiabetic drug).

	Pharmacokinetics						Drug-Likeness		Lead-Likeness (Number of Violations)
Peptide	P-gp Substrate	CYP1A2 Inhibitor	CYP2C19 Inhibitor	CYP2C9 Inhibitor	CYP2D6 Inhibitor	CYP3A4 Inhibitor	Lipinski (Number of Violations)	Abbot Bioavailability Score	
AK	No	No	No	No	No	No	Yes (0)	0.55	No (2)
AY	No	No	No	No	No	No	Yes (0)	0.55	Yes (0)
CF	No	No	No	No	No	No	Yes (0)	0.55	Yes (0)
DL	No	No	No	No	No	No	Yes (0)	0.56	No (2)
EF	No	No	No	No	No	No	Yes (0)	0.56	No (1)
GIL	No	No	No	No	No	No	Yes (0)	0.55	No (1)
GM	No	No	No	No	No	No	Yes (0)	0.55	No (1)
IAL	No	No	No	No	No	No	Yes (0)	0.55	No (1)
IL	No	No	No	No	No	No	Yes (0)	0.55	No (2)
SL	No	No	No	No	No	No	Yes (0)	0.55	No (1)
TF	No	No	No	No	No	No	Yes (0)	0.55	Yes (0)
TY	No	No	No	No	No	No	Yes (0)	0.55	Yes (0)
VF	No	No	No	No	No	No	Yes (0)	0.55	Yes (0)
VL	No	No	No	No	No	No	Yes (0)	0.55	No (1)
VY	No	No	No	No	No	No	Yes (0)	0.55	Yes (0)
Captopril	No	No	No	No	No	No	Yes (0)	0.56	No (1)
Anagliptin	Yes	No	No	No	No	No	Yes (0)	0.55	No (2)

## Data Availability

The data presented in this study are available on request from the corresponding author.
